# RETRACTED: Dukenbayev et al. Fe_3_O_4_ Nanoparticles for Complex Targeted Delivery and Boron Neutron Capture Therapy. *Nanomaterials* 2019, *9*, 494

**DOI:** 10.3390/nano15201590

**Published:** 2025-10-17

**Authors:** Kanat Dukenbayev, Ilya V. Korolkov, Daria I. Tishkevich, Artem L. Kozlovskiy, Sergey V. Trukhanov, Yevgeniy G. Gorin, Elena E. Shumskaya, Egor Y. Kaniukov, Denis A. Vinnik, Maxim V. Zdorovets, Marina Anisovich, Alex V. Trukhanov, Daniele Tosi, Carlo Molardi

**Affiliations:** 1 School of Engineering, Nazarbayev University, 010000 Nur-Sultan, Kazakhstan; kdukenbayev@nu.edu.kz (K.D.); daniele.tosi@nu.edu.kz (D.T.); carlo.molardi@nu.edu.kz (C.M.); 2 The Institute of Nuclear Physics, 050032 Almaty, Kazakhstan; korolkovelf@mail.ru (I.V.K.); kozlovskiy.a@inp.kz (A.L.K.); gorineg@mail.ru (Y.G.G.); mzdorovets@gmail.com (M.V.Z.); 3 L.N. Gumilyov Eurasian National University, 010008 Nur-Sultan, Kazakhstan; 4 Laboratory of Magnetic Films Physics, Cryogenic Research Department, Scientific-Practical Materials Research Centre, National Academy of Sciences of Belarus, 220072 Minsk, Belarus; sv_truhanov@mail.ru (S.V.T.); shumska.alena@gmail.com (E.E.S.); ka.egor@mail.ru (E.Y.K.); truhanov86@mail.ru (A.V.T.); 5 Laboratory of Single crystal growth, South Ural State University, 454080 Chelyabinsk, Russia; vinnikda@susu.ru; 6 Department of Electronic Materials Technology, National University of Science and Technology MISiS, 119049 Moscow, Russia; 7 Ural Federal University named after the First President of Russia B.N. Yeltsin, 620075 Yekaterinburg, Russia; 8 Republican Unitary Enterprise “Scientific-Practical Centre of Hygiene”, 220012 Minsk, Belarus; m_anisovich@mail.ru

This journal retracts the article “Fe_3_O_4_ Nanoparticles for Complex Targeted Delivery and Boron Neutron Capture Therapy” [1], which is cited above.

Following publication, concerns were brought to the attention of the Editorial Office regarding an overlap of images between this article and a previously published article [2] from similar authorship groups.

Adhering to our standard procedure, an investigation was conducted by the Editorial Office and Editorial Board that identified indications of inappropriate editing and duplication between figures presented in this article [1] and an earlier publication [2]. While the authors cooperated during the investigation by providing raw materials and clarifying the availability of the underlying data, the Editorial Board was unable to verify the integrity of the study’s central findings based on the materials provided. As a result, the Editorial Board has lost confidence in the validity of the findings and has decided to retract the paper as per MDPI’s retraction policy (https://www.mdpi.com/ethics#_bookmark30).

This retraction was approved by the Editor-in-Chief of the journal *Nanomaterials*.

The authors did not agree to this retraction.
